# Effects of storage duration of suspended red blood cells before intraoperative infusion on coagulation indexes, routine blood examination and immune function in patients with gastrointestinal tumors

**DOI:** 10.12669/pjms.39.1.7031

**Published:** 2023

**Authors:** Xiaofei Lan, Yan Chen, Qihua Bi, Weihong Xu, Jun Huang

**Affiliations:** 1 Xiaofei Lan, Department of Transfusion Section, Zhejiang Hospital, 12,Lingyin Road, Hangzhou 310013, Zhejiang Province, P.R. China; 2 Yan Chen, Blood Center of Zhejiang Province, 789, Jianye Road, Hangzhou 310052, Zhejiang Province, P.R. China; 3 Qihua Bi, Department of Transfusion Section, Zhejiang Hospital, 12,Lingyin Road, Hangzhou 310013, Zhejiang Province, P.R. China; 4 Weihong Xu, Department of Transfusion Section, Zhejiang Hospital, 12,Lingyin Road, Hangzhou 310013, Zhejiang Province, P.R. China; 5 Jun Huang, Department of Transfusion Section, Zhejiang Hospital, 12,Lingyin Road, Hangzhou 310013, Zhejiang Province, P.R. China

**Keywords:** Gastrointestinal neoplasms, Suspended red blood cells, Storage duration; Coagulation index, Routine blood test

## Abstract

**Objective:**

To investigate the effect of storage duration of suspended red blood cells (SRBC) before intraoperative infusion on coagulation indexes, routine blood examination and immune function in patients with gastrointestinal (GI) tumors.

**Methods:**

We divided clinical data of one hundred patients with GI tumors who underwent surgical treatment in our hospital into two different groups according to the storage duration of SRBC use for intraoperative infusion. The short-term group (n=50) had patients with SRBC storage durations shorter than two weeks, and the long-term group (n=50) had patients with storage durations longer than two weeks. We compared the coagulation, immune function, routine blood profile, electrolyte levels and adverse reactions assessment results between the two groups.

**Results:**

Compared with before transfusions, the levels of fibrinogen (FIB) and activated partial prothrombin time (APTT) after blood transfusions were higher than those before transfusion (*P*<0.05). The levels of hemoglobin (Hb) and hematocrit (HCT) in the two groups after blood transfusions were also higher than those before transfusion (*P*<0.05). However, the levels of CD4^+^ decreased and those of CD8^+^ increased in both groups after the blood transfusions. In addition, the levels of CD4^+^ and CD4^+^/CD8^+^ in the short-term group were higher than those of the long-term group (*P*<0.05) while the CD8^+^ levels were lower than that of the long-term group (*P*<0.05). After the blood transfusions, the potassium ion (K^+^) levels in the two groups increased, and those in the long-term group were higher than in the short-term group (*P*<0.05). The sodium ion (Na^+^) levels in the two groups increased after the transfusions, and the short-term group had higher levels than the long-term group (*P*<0.05). Finally, the incidence of adverse reactions in the short-term group (4.00%) was lower than that in the long-term group (18.00%) (*P*<0.05).

**Conclusion:**

Intraoperative infusion of SRBC with storage duration longer than two weeks increases the risk of perioperative adverse transfusion reactions, which implies that the storage duration of SRBC should be strictly controlled in clinical practice to reduce the risk of blood transfusion.

## INTRODUCTION

Gastrointestinal (GI) tumors are characterized by the presence of an abdominal mass, intestinal obstruction and bloody stools. Early surgical resection of tumors is an effective treatment.^1^ Radical resection is the first treatment choice, but it renders patients prone to anemia due to massive bleeding, malnutrition and other factors. Intraoperative blood transfusions help prevent postoperative anemia,^2^ and suspended red blood cells (SRBC) intraoperative infusions are usually preferred to treat blood losses during surgery. Studies have shown that the SRBC storage duration may affect the function and morphology of its cells, altering the oxygen carrying capacity of erythrocytes.^3^,^4^

Moreover, SRBC may induce a variety of biological, biochemical and physical changes during storage, weakening the efficacy of blood transfusions and increasing the adverse reaction risks including the risk of non-hemolytic fever or multiple organ failure.^3^,^4^ The cell membrane structure and membrane elasticity of SRBC change and they release bioactive cell vesicles during low-temperature storage. These changes of SRBC membrane may induce transfusion reactions and negative posttransfusion outcomes.^5^

Thus clarifying the impact of different SRBC storage durations on the coagulation function and electrolyte levels in transfused patients is important.^6^,^7^ For this study, we retrospectively analyzed the clinical data of patients with GI tumors who received surgical treatment, and discussed the effect of SRBC storage durations on their coagulation indexes, routine blood profile and immune function results to provide a reference for related surgical blood transfusions.

## METHODS

We retrospectively selected clinical data of one hundred patients with GI tumors who underwent surgery in our hospital from February 2018 to February 2022. The short-term group included 50 patients with intraoperative SRBC infusion after a storage duration shorter than two weeks (26 men and 24 women, aged 37-74 years, mean age at 56.76 ± 8.86 years); the long-term group included 50 patients with SRBC storage durations before the intraoperative infusion longer than two weeks (30 men and 20 women, aged 37-71 years, mean age at 58.18 ± 7.12 years). The general data on gender and age were similar between the two groups (*P*>0.05). All patients received enteral and parenteral nutritional support supplemented with glucose and vitamins one day after the operation, and all clinical effects were recorded.

### Inclusion criteria:

- Patients with early stage GI tumors diagnosed by clinic pathology undergoing successful radical tumor resection
- Patients with intraoperative blood loss ≥800 mL and infused SRBC
- Patients without fever or infections and with normal electrolyte, coagulation function indexes, routine blood levels and immune function indexes before the blood transfusion
- Patients with complete clinical data

### Exclusion criteria:

- Patients with immune or blood disorders diseases or with other malignant tumors
- Patients with severe heart, lung, kidney or other functional organ damages
- Patients who had received systemic chemotherapy, immunosuppressant’s or glucocorticoids before the operation
- Patients with a history of prior blood transfusion
- Patients with incomplete immune profile results

### Ethical Approval:

The medical ethics committee of our hospital approved this study (Approval number 2018-C-53, Date: 2018-07-27).

### Observational indicators:

(1) Coagulation function indexes [fibrinogen (FIB), prothrombin time (PT), and activated partial thromboplastin time (APTT): 3-ml fasting venous blood samples were collected and analyzed in a BC-20 automatic blood cell analyzer (Shenzhen Mind ray Biomedical Electronics). (2) Routine blood levels [hemoglobin (Hb), hematocrit (HCT), platelet count (PLT)]: 3-ml fasting venous blood samples were collected and analyzed using a Shenzhen Mind ray CAL8000 blood analysis line system. (3) Immune function indexes [CD4 cells (CD4^+^), CD8 cells (CD8^+^)]: 3-ml fasting venous blood samples were collected and centrifuged for 15 minutes at 3000 rpm (centrifugation radius, 12.5cm), the sera were separated and analyzed by flow cytometry (Taizhou Huamai Medical Instrument) to calculate CD4^+^/CD8^+^ counts (the test kits used were purchased from Shanghai enzyme Biotechnology). (4) Levels of electrolytes [calcium ion (Ca^2+^), potassium ion (K^+^), sodium ion (Na^+^)]: 3-ml fasting venous blood samples were collected and analyzed in an hc-9886 electrolyte analyzer (Shenzhen Hangchuang Medical Equipment). (5) Adverse reactions: treating physicians recorded the occurrence of non-hemolytic fever and allergic transfusion reactions in the patients.

### Statistical Analysis:

We analyzed the data using the SPSS 26.0 statistical software using α=0.05 as a test standard. All measurement data are presented as mean plus standard deviations (*χ̄*±*S*) and compared using t-tests. The counting data are presented as numbers and percentages n, (%) and tested applying a χ^2^ test.

## RESULTS

There were no signifiwcant differences in FIB, PT and APTT between the short-term and long-term groups, whether before or after blood transfusions (*P*>0.05). Compared with before blood transfusion, the levels of FIB and APTT after blood transfusion in each group were higher than those before transfusion, and the differences were statistically significant (P<0.05); however, the PT counts were similar before and after blood transfusions in each group (*P*>0.05). Table-I

**Table-I:** Comparison of coagulation function indexes between the two groups (*χ̅*±*S*)

Group	n	FIB (g/L)		PT (s)		APTT (s)	

		Before blood transfusion	24 hours after blood transfusion	Before blood transfusion	24 hours after blood transfusion	Before blood transfusion	24 hours after blood transfusion
Short-term group	50	1.61±0.34	2.90±0.54^a^	14.96±2.63	15.46±2.81	33.06±4.16	38.41±5.38^a^
Long-term group	50	1.63±0.36	2.85±0.51^a^	14.80±2.57	15.69±2.76	33.29±4.20	38.71±5.42^a^
t	-	-0.286	0.476	0.308	-0.413	-0.275	-0.278
p	-	0.776	0.635	0.759	0.681	0.784	0.782

***Note:***

a indicates a comparison with the same group before the blood transfusion, *p* <0.05.

The levels of Hb, HCT and PLT at the same time points were all similar between the two groups (*P*>0.05). The PLT levels before and after the transfusions were similar in both groups (*P*>0.05). However, the levels of Hb and HCT after the blood transfusions were higher than those before the transfusions (*P*<0.05). Table-II

**Table-II:** Comparison of routine blood profile results between the two groups (*χ̅*±*S*)

Group	n	Hb (g/L)		HCT (%)		PLT (×10^9^/L)	

		Before blood transfusion	24 hours after blood transfusion	Before blood transfusion	24 hours after blood transfusion	Before blood transfusion	24 hours after blood transfusion
Short-term group	50	64.28±5.74	92.37±8.29^a^	20.94±2.33	25.96±3.12^a^	202.14±26.35	196.24±24.18
Long-term group	50	64.71±5.70	91.68±8.16^a^	20.80±2.30	25.70±3.06^a^	203.81±26.26	198.48±24.60
t	-	-0.376	0.419	0.302	0.421	-0.317	-0.459
p	-	0.708	0.676	0.763	0.675	0.752	0.647

***Note:***

a indicates a comparison with the same group before the blood transfusion, *p* <0.05.

Before blood transfusions, there were no significant differences in levels of CD4^+^, CD8^+^, and CD4^+^/CD8^+^ between the two groups (*P*>0.05). However, compared with before transfusions, the CD4^+^ levels after transfusion decreased while the CD8^+^ levels increased in each group. In addition, after the transfusions, the levels of CD4^+^ and CD4^+^/CD8^+^ in the long-term group were lower than those in the short-term group, while the CD8^+^ levels in the long-term group were higher than those in the short-term group (*P*<0.05). Table-III

**Table-III:** Comparison of immune function results between the two groups (*χ̅*±*S*).

Group	n	CD4^+^ (%)		CD8^+^ (%)		CD4^+^/CD8^+^	

		Before blood transfusion	24 hours after blood transfusion	Before blood transfusion	24 hours after blood transfusion	Before blood transfusion	24 hours after blood transfusion
Short-term group	50	35.25±5.30	32.28±5.62	31.32±2.71	34.31±2.94^a^	1.13±0.18	0.94±0.13
Long-term group	50	35.57±5.32	28.25±5.11	31.50±2.74	37.16±3.05^a^	1.12±0.17	0.76±0.11
t	-	-0.301	3.752	-0.330	-4.757	0.286	7.474
p	-	0.764	<0.001	0.742	<0.001	0.776	<0.001

***Note:***

a indicates a comparison with the same group before the blood transfusion, *p* <0.05.

Before blood transfusions, there were no significant differences in levels of Ca^2+^, K^+^, and Na- between the two groups (*P*>0.05). After the transfusions, the levels of Ca^2+^ decreased in both groups, but there were no significant differences in Ca^2+^ levels between the two groups after transfusion (*P*>0.05). The level of K^+^ in both groups increased, and the long-term group had higher levels than the short-term group (*P*<0.05). The Na^+^ level in both groups increased, and the short-term group displayed higher levels than the long-term group (*P*<0.05. Table-IV.

**Table-IV:** Comparison of electrolyte levels between the two groups (mEq/L,*χ̅*±*S*)

Group	n	Ca^2+^		K^+^		Na^+^	

		Before blood transfusion	24 hours after blood transfusion	Before blood transfusion	24 hours after blood transfusion	Before blood transfusion	24 hours after blood transfusion
Short-term group	50	2.17±0.21	1.63±0.36^a^	4.08±0.67	4.49±0.51^a^	128.24±2.34	137.25±3.69^a^
Long-term group	50	2.19±0.22	1.66±0.34^a^	4.13±0.64	4.82±0.54^a^	128.50±2.38	133.38±3.28^a^
t	-	-0.465	-0.428	-0.382	-3.142	-0.551	5.543
p	-	0.643	0.669	0.704	0.002	0.583	<0.001

***Note:***

a indicates a comparison with the same group before the blood transfusion, p <0.05.

The incidence of adverse reactions in the short-term group (4.00%) was lower than that in the long-term group (18.00%) with a significant difference between them (*P*<0.05. Table-V

**Table-V:** Comparison of adverse reactions between the two groups (n%).

Group	n	Non hemolytic fever	Allergic transfusion reaction	Incidence rate
Short-term group	50	1 (2.00)	1 (2.00)	2 (4.00)
Long-term group	50	5 (10.00)	4 (8.00)	9 (18.00)
χ^2^	-			5.005
p	-			0.025

## DISCUSSION

GI tumor is one of the five most common cancers in both men and women which accounts for 26% of the global cancer incidence.^8^,^9^ Surgical resections of GI tumors in patients who meet the surgical indications have a good therapeutic effect. However, it is reported that the surgical treatment cause large tissue traumas and intraoperative blood losses, which may lead to anemia, malnutrition and poor immunity.^10^ Blood transfusion is an effective means to improve the immune regulation and coagulation functions.^11^,^12^ SRBC infusions are a main type of blood transfusions, which can quickly correct and prevent anemia, and have different effects on the patients’ inflammatory reactions, coagulation functions and electrolyte levels.^13^ Studies show that SRBC are prone to storage damage from red blood cell morphology and hydrodynamic changes that induce changes in the cell membrane and biochemistry during low-temperature storage.^14^, ^15^ Different storage durations have different effects on the coagulation and immune functions.^14^,^15^ In our study, we studied data of patients with GI cancer undergoing radical resection of GI tumors to explore the effects of SRBC storage duration on coagulation indexes routine blood tests and immune function results, to obtain a preliminary reference for optimizing the storage duration of SRBC.

Ichikawa et al^16^ and Riccio et al^17^ found that with the extension of storage periods, SRBC are prone to storage damage, changes in red blood cell-related biomechanics, reduced blood transfusion efficiency, which can seriously cause respiratory distress and other problems. Hashemi Tayer et al. also found that during long-term low-temperature storage, the coagulation function of SRBC stocks is impacted by released cytokines such as interleukin and soluble human leukocyte antigen, which can increase the risk of adverse transfusion reactions such as non-hemolytic fever.^18^ We found supporting evidence with the FIB, APTT, Hb and HCT values of patients in the two groups increasing after the blood transfusions compared with the values before the procedure. Our results also indicate that the storage duration of SRBC has different effects on their functionality, and that SRBC age during storage, probably releasing cell vesicles rich in phosphatidylserine and promoting coagulation. Cell vesicles can increase thrombin in plasma and activate the coagulation system through proteins in the cytoskeleton, probably explaining how SRBC affect coagulation (a promising research direction in the clinic.^19^, ^20^

CD4^+^ is an immune marker expressed mainly by helper T cells. It can assemble with the non-polypeptide region of MHC Class-II molecules, participate in the process of antigen recognition, direct the body against pathogenic microorganisms, and help fight infections.^21^ The surface antigens of lymphocytes are produced during cell differentiation. The specific antigens on the surface of a cell membrane can be detected by specific monoclonal antibodies. As a subgroup of T lymphocytes, CD8^+^ cells can further differentiate and proliferate into effector cells after activation. Increases in CD8^+^ values indicate immune function disorders and cellular immunity inhibition.^22^,^23^ Study has reported that the storage duration had no significant effect on the ratio of CD4^+^/CD8^+^ in erythrocyte suspension.^24^ However, our results showed that after blood transfusions, the levels of CD4^+^ decreased and those of CD8^+^ increased in both groups, and that the CD4^+^/CD8^+^ ratio was larger in the short-term group than that in the long-term group, suggesting that the SRBC stored for more than two weeks had a more negative impact on the immune function of patients with GI tumors than SRBC stored for less than two weeks. Further studies should be conducted to explain the conflicting results of these studies.

After blood transfusions, we found that the K^+^ levels in both groups increased, and the long-term group had higher levels than the short-term group, which may be due to the exosmosis of intracellular K^+^ caused by long-term storage and the increase of K^+^ in the supernatant of SRBC solution.^25^ The Na^+^ levels in both groups increased, and the short-term group had higher levels than the long-term group, which may be related to hemolysis of stored SRBC.^26^ In addition, our study also showed that the incidence of adverse reactions in the short-term group (4.00%) was lower than that in the long-term group (18.00%), which was similar with the study results of Fan et al^27^ and Robinson et al.^28^

### Limitations:

This is a single center study, and we only studied patients with GI tumor surgery. Follow-up studies should address SRBC transfusions under different circumstances in prospective and multi center studies.

## CONCLUSION

Intraoperative infusion of SRBC with storage durations longer than two weeks in patients with GI tumors may increase the risk of adverse transfusion reactions such as allergic transfusion and non-hemolytic fever, which implies that the storage duration of SRBC should be strictly controlled in clinical practice to reduce the risk of blood transfusion.

### Authors’ contributions:

**XL** conceived and designed the study.

**YC, QB, WX and JH** collected the data and performed the analysis.

**XL** was involved in the writing of the manuscript and is responsible for the integrity of the study.

All authors have read and approved the final manuscript.
